# MRI for risk stratification of muscle invasion by upper tract urothelial carcinoma: a feasibility study

**DOI:** 10.1186/s41747-023-00403-3

**Published:** 2024-01-19

**Authors:** Emanuele Messina, Flavia Proietti, Ludovica Laschena, Rocco Simone Flammia, Martina Pecoraro, Stefano Cipollari, Giuseppe Simone, Carlo Catalano, Costantino Leonardo, Valeria Panebianco

**Affiliations:** 1 Department of Radiological Sciences, Oncology and Pathology, Sapienza University/Policlinico Umberto I, Viale del Policlinico 155, Rome, 00185 Italy; 2 Department of Maternal-Infant and Urological Sciences, Sapienza University/Policlinico Umberto I, Viale del Policlinico 155, Rome, 00185 Italy; 3 Department of Urology, IRCCS “Regina Elena” National Cancer Institute, Rome, Italy; 4 Department of Radiological Sciences, Oncology and Pathology, Sapienza University/Policlinico Umberto I, Viale Regina Elena 324, Rome, 00161 Italy

**Keywords:** Cancer staging, Carcinoma (transitional cell), Magnetic resonance imaging, Tomography (x-ray computed), Ureter

## Abstract

**Background:**

Magnetic resonance imaging (MRI) is recommended in patients with upper tract urothelial carcinoma (UTUC) only when computed tomography (CT) is contraindicated. However, CT does not allow distinguishing ureter wall layers, making impossible to assess muscle invasion, a factor contributing to differentiate high- from low-risk UTUCs, which require different therapeutic approaches. We investigated the feasibility of MRI assessment of UTUC muscle invasion.

**Methods:**

From June 2022 to March 2023, we prospectively enrolled patients suspected of UTUC, i.e., with positive urinary tract ultrasound and/or ureteroscopy, or positive urinary cytology and/or hematuria but negative cystoscopy and bladder ultrasound at two Italian centers. They underwent CT followed by MRI (≤ 24 h apart), independently reported by two experienced radiologists, blinded from histopathology results. After imaging confirmation, they all underwent nephroureterectomy and histopathology analysis. Sensitivity, specificity, positive predictive value (PPV), negative predictive value (NPV), accuracy, and area under the receiver operating characteristic curve (AUC) were calculated.

**Results:**

Thirty-nine lesions were detected in 30 patients on both CT and MRI. Muscle-invasive UTUC prevalence was 81% (21/26) among patients with MRI suspicion and 8% (1/13) among those without MRI suspicion (*p* < 0.001). Considering the assessment of muscle-layer invasion, the more experienced reader achieved 95% sensitivity (95% confidence interval 82−100), 71% specificity (47−88), 81% PPV (63−93), 92% NPV (70−100), 85% accuracy (67−96), and 0.84 AUC (0.70−0.98). Inter-reader agreement was substantial (*κ* = 0.73).

**Conclusions:**

MRI showed a promising diagnostic performance for the assessment of UTUC risk of muscle invasion.

**Relevance statement:**

Resulting feasible both in technical and clinical terms, MRI could be helpful for upper tract urothelial carcinomas pre-operative risk stratification, to allow a personalized patients’ management. These results play in favor of promoting preoperative MRI for UTUC, as already proven for bladder cancer.

**Key points:**

• Muscle invasion is a crucial information for tailored treatments of upper tract urothelial carcinomas.

• CT does not distinguish ureter wall layers, making muscle invasion risk assessment not feasible.

• MRI was shown to reliably diagnose muscle-layer invasion by upper tract urothelial carcinomas (sensitivity 95%, specificity 71%).

**Graphical Abstract:**

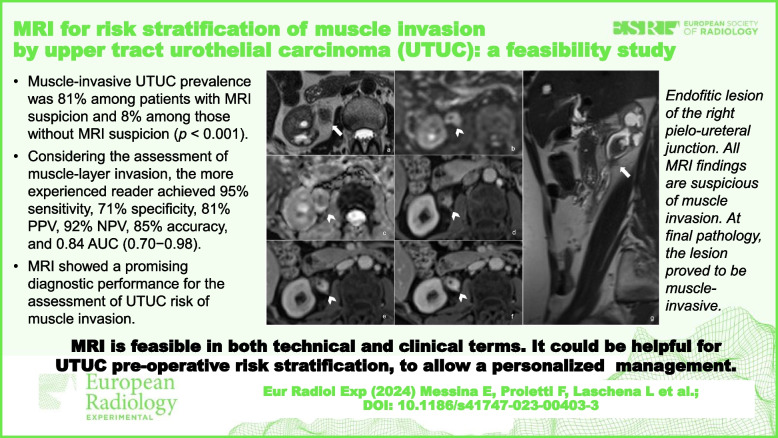

## Background

Urothelial carcinoma represents the fourth most frequent malignancy worldwide, and it can arise from the upper urinary tract, namely from pyelocaliceal cavities or ureter (upper tract urothelial carcinomas, UTUC), or from the lower urinary tract (bladder and urethra). Among urothelial carcinomas, bladder cancers are the most frequent (90−95% of cases), while UTUCs represents the 5−10% [1–3]. UTUC is more frequently invasive at the time of diagnosis (two thirds of cases), compared to bladder cancer (15−25%) [4], and its infiltration of the muscle layer is usually associated to a very poor prognosis, with a 5-year specific survival lower than 50% for pT2/pT3 and than 10% for pT4 [5].

The goal of this research is to focus on UTUC diagnostic workup, and computed tomography (CT) urography proved the highest diagnostic accuracy for its detection [6], while whole-body CT represents the standard of care for initial staging [5]. Conversely, magnetic resonance imaging (MRI) urography is recommended to study patients with UTUC only when CT is contraindicated (i.e., when radiation exposure and/or iodinated contrast media injection are contraindicated) [3, 7], especially because up to now MRI resulted to be inferior to CT for UTUC diagnosis and staging [8]. However, CT cannot properly describe ureter wall layers, making it impossible to assess the suspect of muscle invasion.

It is of note that MRI is powerful in this scenario thanks to the high soft tissue contrast resolution and its capacity in characterizing tumor infiltration of the wall and perivisceral fat tissue or adjacent organ invasion [9]. This has already been described for multiparametric MRI of the bladder, with the design, validation, and spread of vesical imaging-reporting and data system (VI-RADS), which can accurately predict the risk of muscle invasive bladder cancer [10–12]. MRI of the bladder proved its outstanding diagnostic performance in multiple settings, promoting an imaging-based definition of bladder cancer T-stage [9, 13, 14].

**Fig. 1:**
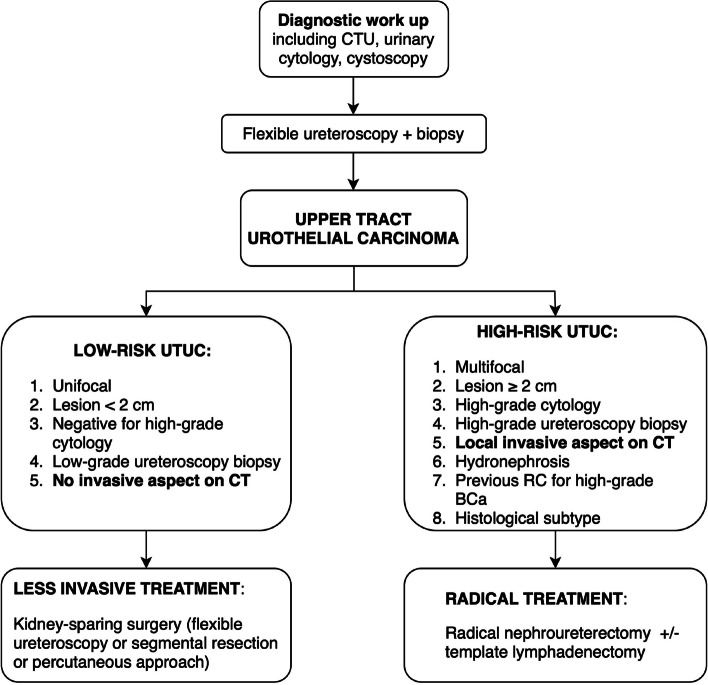
Summary of the updated recommendations on UTUC management; adapted from EAU Guidelines [reference #7]. *BCa*, Bladder cancer; *CTU*, Computed tomography urography; *EAU*, European Association of Urology; *RC*, Radical cystectomy; *UTUC*, Upper tract urothelial carcinoma

Thus, we hypothesized that MRI may prove its efficacy in local staging and characterization of UTUC to a similar extent of what has been already observed in bladder cancers, thanks to their comparability in terms of urothelial origin, macroscopic features, and pathologic staging [3]. Muscle invasion in UTUC is a critical factor because it reflects the advanced stage of the disease, necessitates more aggressive treatments, and is associated with a less favorable prognosis, highlighting the importance of early detection and personalized treatment strategies. Indeed, the nonspecific definition of an invasive aspect on CT is already considered among the coexisting factors differentiating high- from low-risk patients, as indicated in Fig. 1, showing the diagnostic and therapeutic workflow recommended by the European Association of Urology [7]. Indeed, kidney-sparing surgery for low risk UTUCs is now recommended, while for decades, radical nephroureterectomy represented the standard of care [3, 7]. In this context, new imaging tools capable of accurately describing UTUC local staging are needed, to promote informed treatment decisions, to improve patient outcomes, and to face the growing demand for eligible candidates for more conservative treatments. Nonetheless, no previous study investigated the potential role of MRI for local staging of UTUC and for the definition of muscle invasion.

To address this unmet clinical need, we designed the current prospective study, to enhance the use of imaging tools in UTUC staging, with the principle aim of investigating the feasibility of multiparametric MRI to assess the risk of UTUC muscle invasiveness.

## Methods

### Study design and patient population

**Fig. 2:**
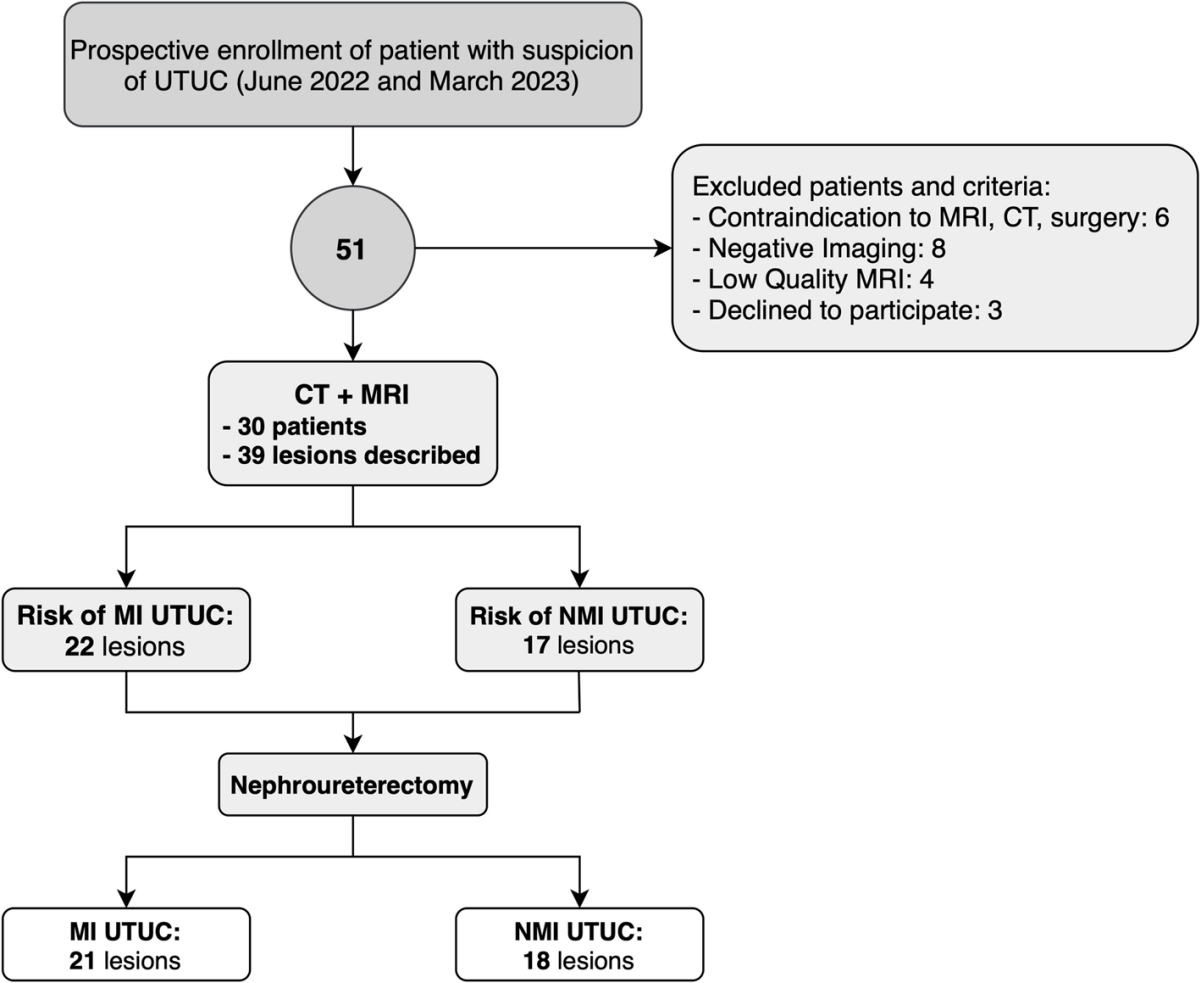
Study flowchart, showing the protocol’s different phases, inclusion and exclusion criteria, and the main outcomes. *CT*, Computed tomography; *MI-UTUC*, Muscle invasive upper tract urothelial carcinoma; *MRI*, Magnetic resonance imaging; *NMI-UTUC*, Non-muscle invasive upper tract urothelial carcinoma

This is a prospective two-center observational study that received formal approval from the Institutional Review Board and the Ethical Committee of our institution (see Declarations). All patients signed a written informed consent. Patients with suspicion of UTUC were referred to two different centers (Sapienza University of Rome [center #1] and “Regina Elena” National Cancer Institute of Rome [center #2]) between June 2022 and March 2023. They were offered a CT scan followed by an MRI no later than 24 h from the CT (both CT and MRI performed in center #1), within the week before surgery (which was performed in centers #1 or #2). Patients with imaging confirmation (based on the more experienced reader’s report) underwent nephroureterectomy (robot-assisted or laparoscopic) and consequent histopathological analysis (Fig. 2).

For patients with more than one lesion, the one showing extravisceral extension or the largest in size was considered as target for the definition of the MRI protocol. In case multiple lesions with equal size, all of them were considered target. To be included, all patients had to be ≥ 18 years old and suspected of having UTUC, because of positive urinary tract ultrasound and/or ureteroscopy, or positive urinary cytology and/or hematuria but negative bladder ultrasound and cystoscopy [3]. The exclusion criteria were history of prior urogenital neoplasms and/or treatments directed to the genitourinary system; any contraindication to CT and/or to MRI (low renal function, MRI unsafe medical devices etc.), to surgery procedures, and to spinal and general anesthesia; active urinary tract infection; and no detectable lesion on CT and/or MRI (Fig. 2).

### CT protocol

CT images were obtained using a 64-row multidetector scanner (Somatom Definition, Siemens Medical Solutions, Forchheim, Germany). A precontrast image acquisition was obtained, followed by the intravenous injection of iodinated contrast media (iomeprol 350 mg/mL, or iopromide 370 mg/mL; 100–120 mL; 3.5–4.0 mL/s). CT urography protocol was divided into three phases: (a) corticomedullary phase (25−35 s after injection), (b) nephrographic phase (80–100 s), (c) excretory phase (10−16 min). CT images were mostly used to assess lesions’ site and consequently to help to define the following MRI protocol. For the purpose of patient management, CT images were exploited to define distant staging. Scanning parameters were as follows: tube voltage 120 kVp; tube current 100–250 mAs; pitch 1.2; and collimation 0.625–0.75 mm. Images were reconstructed using a 1-mm slice thickness on axial and coronal planes, using both soft tissue kernel (B31f) and lung kernel (B75f) reconstruction.

### MRI protocol

Examinations were performed using a 3-T scanner (MAGNETOM Vida, Siemens Medical Solutions, Forchheim, Germany), using a 32-channel surface phased-array coil. The protocol consisted of a first coronal T2-weighted imaging (T2WI) turbo-spin-echo sequence, necessary for plane orientation of the following sequences, which were oriented according to the axis of the segment of the excretory system where the target lesion was located. The protocol included (a) at least two planes (axial coronal, and/or sagittal) high-resolution T2WI with a slice thickness of 3−4 mm; (b) one T2WI with fat saturation; (c) diffusion weighted imaging (DWI) (*b* = 0, 800, and 1,000 s/mm^2^) and apparent diffusion coefficient, (ADC) map; (d) three-dimensional dynamic sequence before and after contrast injection (gadoteric acid; 0.2 mmol/kg of body weight; 3.0–3.5 mL/s), to obtain corticomedullary, nephrographic, and excretory phases at 25–35 s, 80–100 s, and 12 min, respectively; and (e) a final post-contrast T1-weighted sequence. MRI specific parameters are reported in Table 1.

**Table 1:** Parameters of the MRI protocol, using two 3-T scanners

Sequence	Parameters								
	TR (ms)	TE (ms)	Flip angle (degrees)	Field of view (cm)	Matrix	Slice thickness (mm)	Interslice gap (mm)	Number of excitations	*b* values (s/mm^2^)
T2WI	4,690	119	90	22−36	400 × 256−320	3	0−0.6	2−3	–
DWI	2,500−5,300	61	90	22−36	128 × 128	3	0−0.6	4−8	0–800-1,000
DCE	3.8	1.2	15	22−36	192 × 192	1	0	1	–

*DCE* Dynamic contrast-enhancement, *DWI* Diffusion-weighted imaging, *MRI* Magnetic resonance imaging, *T2WI* T2-weighted imaging, *TE* Time of echo, *TR* Repetition time

### Image and specimen analysis

CT and MRI images were independently reported by two radiologists, with 5 and over 15 years of experience in genitourinary imaging. They were both blinded from pathologic tumor stage since imaging was acquired and reported before surgery. The following imaging parameters were described: (a) tumor morphology (sessile or papillary wall thickening); (b) total or incomplete ureteral obstruction; (c) lesion’s dimensions (three diameters); (d) perivisceral fat tissue infiltration (absent, equivocal, or present); (e) muscular layer infiltration (absent, equivocal, or present); (f) hydronephrosis grade (from 0 to 4 [15]); (g) renal atrophy (absent or present); (h) renal infiltration (absent, equivocal, or present); (i) local lymph node involvement (absent, equivocal, or present).

Muscular layer infiltration was defined as focal or diffuse interruption of the hypointense line indicating the muscular layer on T2WI and hyperintensity on DWI and contrast enhancement involving the entire thickness of the wall, with the presence of risk of muscle invasion defining a T2 radiological stage. Perivisceral fat invasion was defined as “fat stranding” appearance on T2WI, with extension of the intermediate signal intensity tissue to perivisceral fat, associated with extension of the hyperintensity on DWI and contrast enhancement thorough and beyond the ureteral wall, with the presence of the risk of perivisceral fat invasion defining a T3 radiological stage [10]. Several cases did not meet all the required criteria, showing positive features only in some sequences, and therefore, they were initially indicated as equivocal. In the final analysis, the equivocal cases were considered as positive.

All patients were treated with nephroureterectomy in two tertiary referral high-volume institutions by two experienced urologists (both of them with over 15 years of experience). The surgical specimens were analyzed by a team of experienced genitourinary pathologists (each of them with over 15 years of experience) who reported the number of lesions, the exact lesion location, and the possible presence of periureteral fat invasion and muscle infiltration, as well as surgical tumor grade and stage according to American Joint Committee on Cancer, AJCC, updated recommendations [16, 17].

### Standard of reference and statistical analysis

First, demographics, clinical, radiologic, and pathologic characteristics were reported on either per-patient or per-lesion basis and stratified between non-muscle invasive UTUC (NMI-UTUC) and muscle invasive UTUC (MI-UTUC) (pT < 2 versus pT2−4 at final pathology). Wilcoxon and *χ*^2^ test were used to test differences. Concordance between the two readers in the evaluation of different MRI findings (muscle and perivisceral fat tissue invasion) was calculated using Cohen’s *κ* statistic.

Second, MRI diagnostic performance for the definition of the risk of muscle layer and perivisceral fat tissue invasion was assessed according to both readers, considering final pathology as the reference standard. Specifically, after matching MRI images with histopathological results, MRI findings were considered as true-positive or true-negative if matching with the pathological report. Sensitivity, specificity, positive predictive value (PPV), negative predictive value (NPV), and accuracy were calculated; receiver operating characteristic curves were generated and areas under the curve (AUCs) calculated.

All statistical analyses were performed using Statistical Package for the Social Sciences (SPSS) version 28 (IBM, USA). All tests were two-sided, and statistical significance was set at *p* < 0.05.

## Results

### Baseline characteristics

Overall, thirty patients were included in the final analysis, with a median age of 73 years (interquartile range 67.0−77.5). The majority were male (18/30, 60%), had positive smoking history (20/30, 68%), gross hematuria (23/30, 77%), and positive urinary cytology (17/30, 57%). At final pathology, 39 lesions were identified, with the majority exhibiting pT2−4 stage (21/39, 54%) and high tumor grade (24/39, 61.5%). After stratification of the entire cohort according to muscle invasion at final pathology, a higher proportion of patients with positive urinary cytology (43% versus 13%, *p* = 0.012) as well as higher rates of lesions with MRI suspicion of muscle invasion (54% versus 13%, *p* < 0.001), perivisceral fat infiltration (23% versus 0%, *p* < 0.001), and high tumor grade (41% versus 20%, *p* = 0.042) were recorded between the MI-UTUC versus NMI-UTUC groups, respectively. Renal parenchyma infiltration was suspected in 4/39 cases (10%) both on CT and MRI images, with confirmation at final pathological in every case. The median maximum diameter was 31.9 cm (interquartile range 0.7−12.0). Clinical, radiologic, and pathologic characteristics are summarized in Table 2.

**Table 2:** Summary of cohort population’s clinical, radiological, and pathological data

Variable	Total cohort	NMI-UTUC (< pT2)	MI-UTUC (≥ pT2)	*p*-value*
**Clinical features (per patient)**				
Sample size, *n* (%)	30 (100)	13 (43.3)	17 (56.7)	-
Age, years, median (IQR)	73 (67–77.5)	70.0 (67–74)	74 (67–81)	0.602
Gender, *n* (%)				
Female	12 (40.0)	5 (16.7)	7 (23.3)	0.880
Male	18 (60.0)	8 (26.7)	10 (33.3)	
Positive smoking history, *n* (%)	20 (66.7)	9 (30.0)	11 (36.7)	0.794
Body mass index (kg/m^2^), median (IQR)	24.5 (23.0–27.7)	25.7 (24.8–27.5)	23.4 (22.2–27.7)	0.127
Presence of hematuria, *n* (%)	23 (76.7)	9 (30.0)	14 (46.7)	0.400
Positive urine cytology, *n* (%)	17 (56.6)	4 (13.3)	13 (43.3)	**0.012**
Creatinine (mg/dL), median (IQR)	1.1 (0.9–1.5)	1.4 (1.0–1.7)	1.1 (0.9–1.4)	0.346
Hemoglobin (ng/mL), median (IQR)	12.8 (11.9–14.2)	13.1 (12.6–14.3)	12.5 (11.9–13.2)	0.225
Presence of multifocal diseases, *n* (%)	14 (46.7)	6 (20.0)	8 (26.7)	0.961
**Radiological features (per lesion)**				
Sample size, *n* (%)	39 (100)	17 (43.6)	22 (56.4)	-
Morphology				
Sessile, *n* (%)	36 (92.3)	16 (41.0)	20 (51.3)	0.210
Papillary, *n* (%)	3 (7.7)	1 (2.6)	2 (5.1)	
MR suspicion of muscle invasion, *n* (%)				
Yes	26 (66.7)	5 (12.8)	21 (53.8)	**< 0.001**
No	13 (33.3)	12 (30.8)	1 (2.6)	
Excretory tract stenosis *n* (%)				
No	8 (20.5)	2 (5.1)	6 (15.4)	0.178
Present, < 50%	7 (17.9)	5 (12.8)	2 (5.1)	**0.020**
Present, ≥ 50%, < 75%	9 (23.1)	4 (10.3)	5 (12.8)	0.907
Present, ≥ 75%	15 (38.5)	6 (15.4)	9 (23.1)	0.542
MR suspicion of perivisceral fat infiltration, *n* (%)				
Yes	9 (23.1)	0 (0)	9 (23.1)	**< 0.001**
No	30 (76.9)	30 (76.9)	0 (0)	
Adjacent organs infiltration, *n* (%)	1 (2.6)	0 (0)	1 (2.6)	0.348
Hydronephrosis, *n* (%)				
No	10 (25.6)	5 (12.8)	5 (12.8)	0.777
1 grade	7 (17.9)	3 (7.7)	3 (7.7)	0.847
2 grade	9 (23.1)	3 (7.7)	7 (17.9)	0.159
3 grade	10 (25.6)	4 (10.3)	6 (15.4)	0.308
4 grade	3 (7.6)	2 (5.1)	1 (2.6)	0.643
Kidney atrophy, *n* (%)	4 (10.3)	2 (5.1)	2 (5.1)	0.871
MR suspicion of nodes involvement, *n* (%)	9 (23.1)	1 (2.6)	8 (20.5)	**< 0.001**
**Surgical and pathological features (per lesion)**				
Sample size, *n* (%)	39 (100)	18 (46.2)	21 (53.8)	-
Primary tumor stage (pT)				
0	1 (2.6)	1 (2.6)	-	**-**
A	6 (15.4)	6 (15.4)	-	
IS	1 (2.6)	1 (2.6)	-	
1	10 (25.6)	10 (25.6)	-	
2	12 (30.8)	-	12 (30.8)	
3	8 (20.5)	-	8 (20.5)	
4	1 (2.6)	-	1 (2.6)	
Tumor grade				
Low	15 (38.5)	10 (25.6)	5 (12.8)	**0.042**
High	24 (61.5)	8 (20.5)	16 (41.0)	

The most experienced radiologist’s reports were considered to design this table. *p-value < 0.05 was considered for statistical significance (significant values in bold). *IQR*, Interquartile range; *MI-UTUC*, Muscle-invasive upper tract urothelial carcinoma; *NMI-UTUC*, Non-muscle-invasive upper tract urothelial carcinoma; *MR*, Magnetic resonance

### MRI diagnostic performance in the assessment of UTUC muscle invasion

**Fig. 3:**
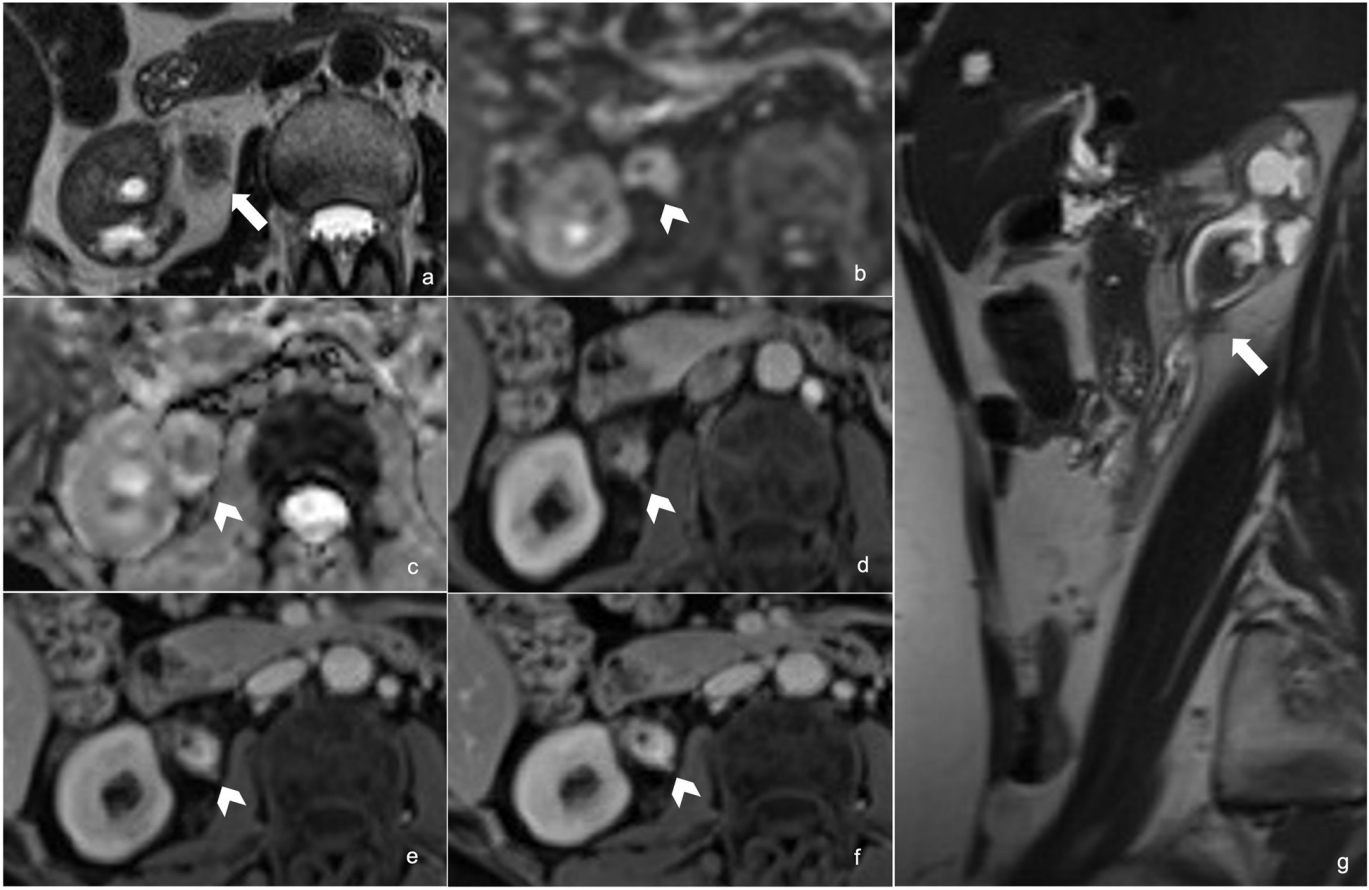
A 64-year-old female patient presenting with gross hematuria and neoplastic cells at urine cytology. **a**, **g** T2WI (axial and sagittal plane) shows an endophytic lesion (13 × 20 mm) of the right pyelo-ureteral junction (white arrow), which determines stenosis of the lumen, third-grade hydronephrosis on the same side and adjacent “fat stranding” appearance. **b**, **c** DWI (*b* = 1,000 s/mm^2^) and ADC map show a lesion with a significant restriction of diffusion (white arrowhead). **d**, **e**, **f** Dynamic sequences after gadolinium-based contrast injection (corticomedullary, nephrographic, and excretory phase) show an early lesion enhancement, extending through the muscularis layer (white arrowhead). All these MRI findings are suspicious of muscle invasion. At final pathology, the lesion proved to be muscle-invasive. *ADC*, Apparent diffusion coefficient; *DWI*, Diffusion-weighted imaging; *T2WI*, T2-weighted imaging

**Fig. 4:**
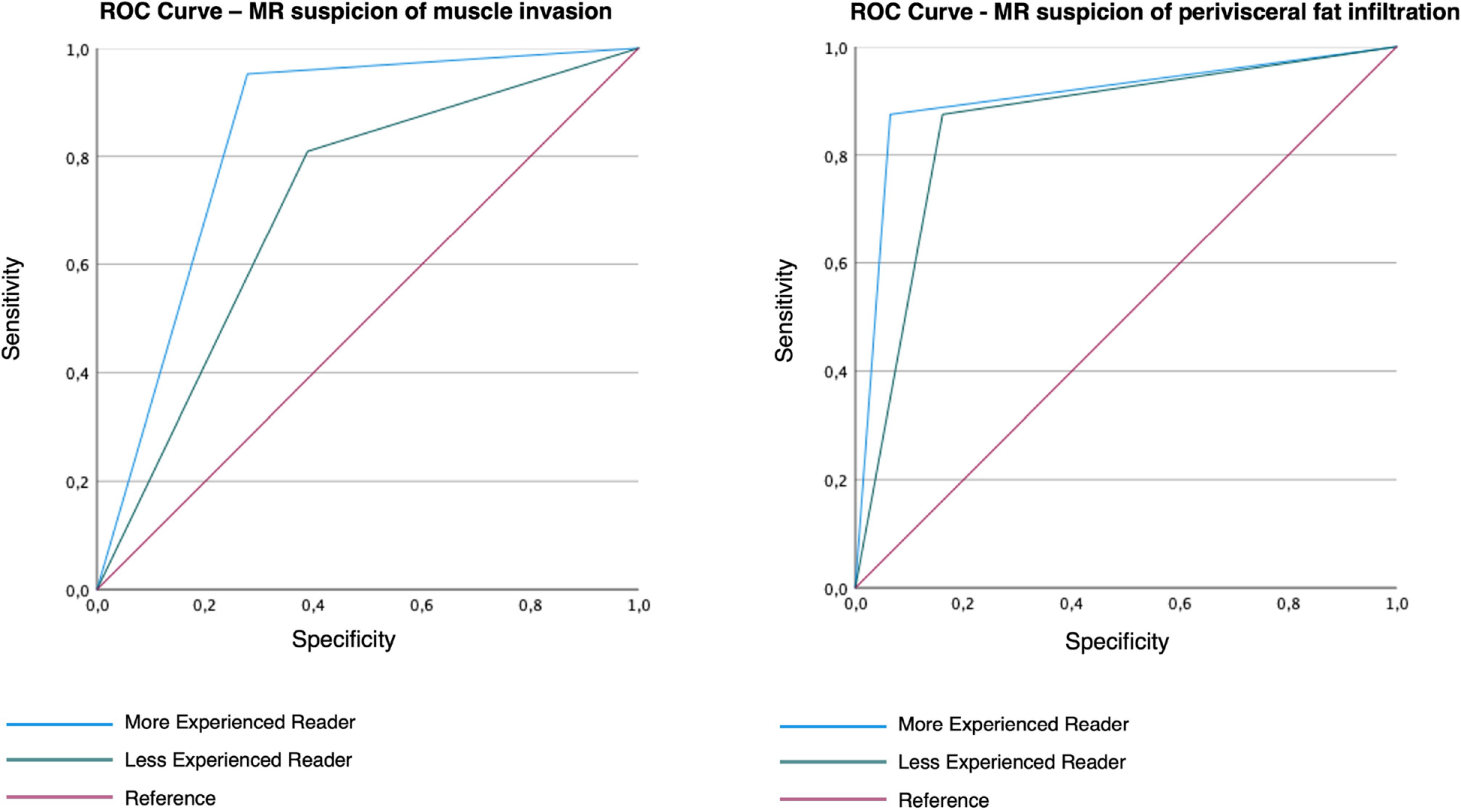
Receiver operating characteristic analysis for the performance of MRI, in detecting UTUC muscle-layer and perivisceral fat tissue invasion, for both the more and the less experienced readers. *MRI*, Magnetic resonance imaging; *UTUC*, Upper tract urothelial carcinoma

When considering the definition of the risk of UTUC muscle invasion on MRI, the inter-reader agreement was substantial (*κ* = 0.73). According to the most experienced reader, MRI suspicion of muscle layer invasion was reported for 26/39 lesions (67%). The prevalence of pathology proven MI-UTUC among patients with MRI suspicion of muscle invasion was 81% (21/26) (Fig. 3), while it resulted 8% (1/13) among those without MRI suspicion (*p* < 0.001). The most experienced reader described 5/39 (13%) equivocal lesions, while the less experienced 7/39 (18%). DCE was the sequences showing the higher rate of equivocal pattern (5/5 cases for the most experienced reader and 7/7 for the less experienced). For the most experienced reader, MRI showed a per-lesion sensitivity, specificity, PPV, NPV, and accuracy of 95%, 71%, 81%, 92%, and 85%, respectively, with an AUC of 0.84 (95% confidence interval 0.70−0.98). The diagnostic performance analysis results, also considering the less experienced reader, are summarized in Table 3, and receiver operating characteristic curves are showed in Fig. 4.

**Table 3:** Per-lesion diagnostic performance and area under the curve of MRI analyzed by both the more and the less experienced readers for the definition of UTUC infiltration of muscular layer and perivisceral fat tissue

		Sensitivity % (95% CI)	Specificity % (95% CI)	PPV % (95% CI)	NPV % (95% CI)	Accuracy % (95% CI)	AUC (95% CI)
UTUC muscle invasion	More experienced reader	95 (82–100)	71 (47–88)	81 (63–93)	92 (70/100)	85 (67–96)	0.84 (0.70−0.98)
	Less experienced reader	81 (61–94)	61 (38–81)	71 (51–86)	73 (48–91)	72 (55–83)	0.71 (0.54−0.88)
UTUC infiltration of perivisceral fat tissue	More experienced reader	87 (56–99)	93 (81–99)	78 (46–96)	97 (86–100)	92 (72–100)	0.91 (0.76−1.00)
	Less experienced reader	78 (46–96)	87 (72–96)	64 (35–87)	93 (80–99)	85 (69–97)	0.86 (0.70−1.00)

*AUC* Area under the curve, *CI* Confidence interval, *MRI* Magnetic resonance imaging, *NPV* Negative predictive value, *PPV* Positive predictive value, *UTUC* Upper tract urothelial carcinoma

### MRI diagnostic performance in the assessment of UTUC infiltration of perivisceral fat tissue

When considering the risk of perivisceral fat tissue infiltration, the inter-reader agreement resulted almost perfect (*κ* = 0.81). For the most experienced reader, MRI suspicion of perivisceral fat tissue invasion was set on 9/39 lesions (23%). The prevalence of pathology-proven perivisceral fat infiltration among patients with MRI suspicion was 78% (7/9), while it resulted 3% (1/30) among those without MRI suspicion (*p* < 0.001).

MRI showed a per-lesion sensitivity, specificity, PPV, NPV, and accuracy of 87%, 93%, 78%, 97%, and 92%, respectively, for the most experienced reader, who achieved an AUC of 0.91 (95% confidence interval 0.76−1.00). The diagnostic performance analysis results, also considering the less experienced reader, are summarized in Table 3, and receiver operating characteristic curves are showed in Fig. 4.

## Discussion

The present experience highlights MRI’s promising results in terms of definition of UTUC perivisceral fat infiltration and especially muscle-layer involvement. Albeit preliminary, in the form of feasibility study, they show an interesting potential role of MRI in UTUC diagnostic work up and T-stage definition.

UTUCs with periureteral fat infiltration are indicated as T3 stage and associated with an increased risk of recurrence after surgery. As a result, it is widely recognized that preoperative prediction of periureteral fat invasion can be crucial for treatment strategy planning [5, 18]. There are only a few data in literature describing the performance of imaging tools for T-staging of UTUC. For example, Honda et al. [19] analyzed CT urography performance founding a sensitivity of 88% and a specificity of 93% for the identification of pT3 and higher UTUCs. Moreover, a meta-analysis comparing more than 1200 patients revealed a pooled sensitivity of CT urography of 92% and a pooled specificity of 95% [6]. On the other hand, Takahashi et al. [8] investigated MRI-urography capability of generally detecting UTUC, obtaining a sensitivity of 75%.

Nonetheless, the present study is the first one expanding imaging threshold not only to the definition of T3 stage but also of T2 stage. Indeed, the most experienced reader achieved an excellent diagnostic performance in assessing the risk of UTUC muscle invasion, with very high accuracy and AUC. The agreement between the two readers resulted substantial. The performance of the less experienced reader was slightly weaker compared to the other, likely because of the absence of a standardized reporting approach. Specificity and PPV were lower compared to sensitivity and NPV probably because we considered the equivocal cases as positive in the final analysis, with the aim of reducing the rate of missed muscle invasive lesions. As a novel application of MRI, radiologists should emphasize the ambiguous presentation of these lesions in clinical practice. This will serve as a signal to alert surgeons, prompting them to meticulously evaluate different treatment approaches.

Due to intrinsic characteristics, MRI can describe the risk of muscle invasion (T2 stage), while CT cannot do that [20]. Therefore, evaluating these results as a whole, MRI could be considered as innovative imaging tool for UTUC local staging, furnishing relevant information, being also a radiation-free, minimally invasive imaging modality.

To the best of our knowledge, these results shed a new light on local staging of UTUC, and, once replicated in larger studies, they could support a relevant change in diagnostic workup and treatment planning of UTUC. In fact, biopsy and urinary cytology, which are included in risk stratification, are often not diagnostic [21]. A precise MRI description of the muscle layer infiltration could be added to the factors distinguishing high- from low-risk patients, in addition to the generic and insufficient definition of “local invasion on CT” as defined in the European Association of Urology guidelines [7]. An accurate estimation of muscle invasion may help urologists to identify patients who could be directed to kidney-sparing treatment, as indicated in Fig. 1 [7]. Moreover, the precise identification of perivisceral fat infiltration may help clinicians to select ideal candidates for neoadjuvant systemic therapy. To date, due to the lack of standardized and accurate preoperative staging, current guidelines recommend adjuvant therapy instead of neoadjuvant therapy based on high-risk features at final pathology [3]. The use of MRI for UTUC local staging may encourage urologists to propose neoadjuvant therapy in cases selected by using MRI, with potential advantages including better response to surgery, positive impact on renal functional reserve, and better preoperative performance status.

Despite its novelty and potential implications, our study is not devoid of limitations. First, we relied on a limited sample size but still representative of this rare disease. Second, the MRI protocol was set on target lesions; consequently, the other lesions in multifocal cases were described on not specifically oriented MRI images with the exception of multiple lesions with equal size. However, our results show how the overall diagnostic performance was not too badly influenced and prove that in clinical practice the definition of the MRI protocol on only target lesion may be sufficient. Finally, CT and MRI scans were performed in close succession, without sufficient time for memory extinction to ensure that the reporting of the first did not influence the second one, especially considering perivisceral fat infiltration. Nevertheless, the main purpose of our study was to primarily evaluate UTUC muscle invasiveness, not the superiority of MRI in identifying perivisceral fat infiltration.

In conclusion, our results showed that MRI is feasible both in technical and clinical terms, showing a potential role in UTUC diagnostic workup: after localization of UTUC lesions on CT urography images, multiparametric MRI could assess the risk of muscle invasion and perivisceral fat infiltration. Furthermore, these results could endorse the MRI protocol adopted here in future larger studies, needed to introduce MRI in the pretreatment definition of UTUC stage.

## Data Availability

The datasets used and analyzed during the current study are available from the corresponding author on reasonable request.

## Abbreviations

AUC: Area under the receiver operating characteristic curve
CT: Computed tomography
DWI: Diffusion weighted imaging
MI-UTUC: Muscle invasive upper tract urothelial carcinoma
MRI: Magnetic resonance imaging
NMI-UTUC: Non-muscle invasive upper tract urothelial carcinoma
NPV: Negative predictive value
PPV: Positive predictive value
T2WI: T2-weighted imaging
UTUC: Upper tract urothelial carcinomas
